# Does *Daucus carota* L. leaf provide a high potential as a source of bioactive constituents: A case study about the influences of process/storage conditions

**DOI:** 10.1002/fsn3.4232

**Published:** 2024-05-27

**Authors:** Merve Seçil Bardakçi, Ayşe Özçelik, Erkan Karacabey

**Affiliations:** ^1^ Department of Food Engineering Suleyman Demirel University Isparta Turkey

**Keywords:** antioxidant capacity, ascorbic acid content, carrot leaves, *Daucus carota* L., drying treatments, phenolic content

## Abstract

The current study focused on the valorization of carrot leaves, *Daucus carota* L. because of their high amount of ascorbic acid (AA), phenolic compounds, and the related antioxidant activity. In this study, the changes in carrot leaves caused by different drying techniques (freeze, vacuum, microwave‐assisted infrared, oven) and different storage conditions (room temperature and refrigerator) were investigated. AA contents of fresh, dried, and stored carrot leaf samples were chromatographically determined. Additionally, analysis of TPA (total phenolic content), TAC (total antioxidant capacity), total chlorophyll, carotenoid, and color were carried out. Additionally, fresh leaves were analyzed to compare their results with the corresponding values of processed or stored samples. TPA, TAC, AA, total chlorophyll, and carotenoid values of the samples stored in the refrigerator were 889 ± 63 mg/100 g d.b., 504 mg/100 g d.b., 269 A.A./100 g d.b., 253 mg/100 g d.b., and 2497 mg/100 g d.b., respectively, while the values of the samples dried at room temperature 620 ± 35 mg/100 g d.b., 303 ± 15 mg/100 g d.b., 110 ± 21 mg A.A./100 g d.b., 44 ± 3 mg/100 g d.b., 641 ± 16 mg/100 g d.b., respectively. Consequently, fresh carrot leaves have a higher vitamin C content than many leafy vegetables and even its own family, parsley. Fresh samples stored at room temperature and in the refrigerator for 7 days provided high ascorbic acid retention. Dried leaves with the MW + IR combined system provided better protection than others in terms of bioactive components. However, OD treatment at 40°C provided better protection and was one step ahead in terms of AA content.

## Graphical abstract

Focused on the valorization of carrot leaves, *Daucus carota* L. because of their high amount of ascorbic acid (AA), phenolic compounds, and the related antioxidant activity. Carrot leave is a promising source of functional constituents, including vitamin C. MW‐IR technique has been demonstrated as a superior method for drying carrot leaves. Utilization of carrot leave powder as a value‐added product is required instead of agriculture waste.

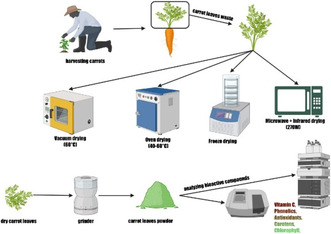

## 1 INTRODUCTION

Rapid processing of fruits and vegetables' green leaves, which are often considered waste, is essential to evaluate and add value to their high bioactive (Raja et al., 2019; Shyamala et al., 2005; Viña & Chaves, 2008). Although fresh leaves have limitations such as short shelf life, seasonal availability, and long supply periods (Onggo et al., 2019), their use has gained interest in the developing world with processing by some techniques. The selection of the appropriate method to process green leafy vegetables should be seriously considered. It has been reported that processing methods have significant effects on the phytochemical compounds found in green leafy vegetables (Putriani et al., 2022). The most common method of preserving leaves is drying due to some advantages like having longer shelf life, simple storage conditions, and easy transportation (Kakade & Hathan, 2014). The resulting dried and powdered leaves are suitable for versatile use and can be used in salads or herbal teas (Kamel, 2013; Türköz et al., 2022). Carrots (*Daucus carota* L.) are a widely popular vegetable worldwide. As a biennial herbaceous plant belonging to the Apiaceae family, they consist of two parts: the root and the leaf (Que et al., 2019). The root, which is the most nutritious part of the plant and is commonly consumed as a vegetable, has a spicy taste that adds a unique flavor to any dish (Song et al., 2018). However, leaves are typically removed after harvesting and used as animal feed or discarded. According to FAO (2022), the global cultivation area for carrots is 1,128,695 hectares, producing a total of 44,762,859 tons. Thus, it is evident that a significant amount of carrot leaves as waste is inevitable. Literature searches indicated that carrot leaves are an excellent source of phytochemicals, including flavonoids, phenolic compounds, terpenoids, steroids, tannins, carotenoids, and beta‐carotene (Shete & Quadro, 2013). In addition, they contain 18.71% protein, 15.69% fiber, and 3.19% oil (Puspani et al., 2020).

Therefore, carrot leaves are worth to be considered as a valuable source of nutrition instead of forage or waste. At this point there are two ways to utilize carrot leaves; direct consumption or processed before use. In the case of direct consumption, the storage period is crucial and needs to be clearly specified its potential effects on fresh carrot leaves depending on storage conditions. In the second way, process techniques and operating conditions are significant and required to be kept in mind to obtain a high value‐added product.

Pereira et al., (2003) emphasized the presence of high ascorbic acid content in carrot leaves and led the interests to this valuable vitamin and to carrot leaves as a potential source. In that study, the adverse effect of storage periods on the ascorbic acid content of carrot leaves was mentioned (Pereira et al., 2003). Furthermore, ascorbic acid is sensible to most of the processes, especially thermal treatments. Low resistance to oxygen exposure is another handicap during the processes. Other bioactive constituents are also adversely affected by process techniques and conditions.

Thus, this study investigated the impact of various drying methods on the composition of bioactive compounds in carrot leaves. The physical properties were analyzed to determine the changes that occur under different processing conditions. Additionally, the effects of storage periods under different conditions on the bioactive contents and physical properties of fresh leaves were studied.

## 2 MATERIALS AND METHODS

### 2.1 Materials

The fresh leaves (*Daucus carota* L.) used in the drying experiments were supplied from a local carrot producer's field located in Konya. Following cleaning, sorting, and mixing, fresh leaves were directly dried by the proposed methods to the final moisture content level (<10% w.b.) which ensures final product safety in terms of microbiological and biochemical reactions.

### 2.2 Drying methods

In this study, to determine the effect of various drying techniques, four different methods were performed: Hot air oven, vacuum, microwave infrared, and freeze drying. For hot air oven drying method, carrot leaves were spread on drying trays (60 cm × 60 cm) as a single layer (depth of 15–20 mm) and dried under hot air stream (0.89 m/s) at two different temperature levels (40 and 60°C) (Mikrotest, MSD2.50D, Turkey). Drying temperature levels were decided according to literature values indicating that most of the studies were conducted at the temperature range of 40–70°C for leafy vegetables (Shyamala et al., 2005; Viña & Chaves, 2008). For vacuum drying, the carrot leaves were dried in a vacuum oven (Wisd, Wov 30, South Korea) at a temperature of 60°C and under a vacuum of about 80 kPa. For microwave‐assisted infrared drying, a microwave oven including infrared lamp was used (LG, 211TAMA00035, Turkey). Drying was carried out at 270 W power level determined by preliminary studies which showed that burning reactions occurred on carrot leaves when the power level exceeded this level. Freeze drying of carrot leaves was conducted in a lab‐scale freeze dryer (operating at pressure level of 0.8 mbar and condenser temperature of −50°C) (Heto, Drywinner DW 3, Denmark) which took about 48 h. Dried leaf samples were ground and then sieved using a 500‐mesh size steel sieve (Kocintok, Haver&Boecker, Turkey). The final moisture content of the ground leaves obtained from various conditions was recorded. The final moisture content was below 10% for all dried samples. Dried carrot leaves and fresh leaves after storage periods were kept at −18°C until analysis.

### 2.3 Determination of total phenolic content and antioxidant capacity

Extraction was carried out by following the method reported by Leite et al. (2011). Approximately 1 g of ground dried leaf sample was weighed and mixed with 10 mL of methanol on magnetic stirring for 5 h and then the supernatant was evaporated until its final volume was less than 1 mL, and the final volume was completed to 5 mL with methanol. After storage, fresh carrot leaves were similarly processed to obtain its phenolic extract. Fresh carrot leaves were also analyzed using the same procedure to compare its results with processed ones. The total phenolic content of the extract was determined by the method reported by Singleton and Rossi (1965). 40 μL of extract and 2.4 mL of distilled water were mixed and vortexed. Then 0.2 mL of Folin–Ciocalteu solution was added. Subsequently, 0.6 mL of saturated sodium carbonate (Na_2_CO_3_) and 0.76 mL of distilled water were consequently mixed and then incubated for 2 h in dark. Samples' absorbances were measured with a spectrophotometer (T70 + UV/VIS spectrophotometer, PG Instruments, UK) at 765 nm wavelength. The total phenolic content (TPC) of samples was calculated in terms of “mg equivalent gallic acid/g dry matter.” The total antioxidant activity of processed carrot leaves was determined using the ABTS method reported by Re et al. (1999) with modification. ABTS/water solution was prepared as 7 mM. ABTS radical cation (ABTS^•+^) was produced by reacting ABTS stock solution with 2.45 mM potassium persulfate (final concentration) and the mixture was left in the dark at room temperature for 12–16 h. ABTS solution was diluted with ethanol up to absorbance reading of 0.700 (±0.02) at 734 nm. Ten microliters of the extract and 990 μL of ABTS solution were mixed. The absorbance of the final solution (at 734 nm) was read at the beginning and 6 min later. The result was calculated as follows (Equation 1).

(1) $$\%\text{Inhibition}=\left[\frac{A_{0}\;-\;A_{1}}{A_{0}}\right]\times 100$$

*A*_0_ and *A*_1_ were the absorbance readings at the beginning and 6 min later, respectively. Results for antioxidant capacity analysis were calculated as “mg trolox equivalent antioxidant capacity/g dry matter”.

### 2.4 Total chlorophyll and total carotenoid content of carrot leaves

For the determination of total chlorophyll and total carotenoid content, all samples were extracted with 80% (v/v) acetone (1:10 w/v) using a mortar and pestle and centrifuged at 2500 × rpm for 10 min. The supernatant was separated, and its absorbance (A) was measured at 663, 645, and 450 nm with a spectrophotometer (T70 + UV/VIS spectrophotometer, PG Instruments, UK). Chlorophyll *a* (Chl.a), chlorophyll *b* (Chl.b), total chlorophyll (Chl.t), and total carotenoid (Crt.t) concentrations were calculated according to the following equations (Equations (2), (3), (4), (5)) (Arnon, 1949).

(2) $$\mathrm{Chl}.t\;\left(\mathrm{mg}/g\right)=\frac{\left(\left(0.0202\times A_{645}\right)+\left(0.00802\times A_{663}\right)\right)\times 10}{\text{sample weight}}$$

(3) $$\mathrm{Chl}.a\;\left(\mathrm{mg}/g\right)=\frac{\left(\left(0.0127\times A_{663}\right)-\left(0.00269\times A_{645}\right)\right)\times 10}{\text{sample weight}}$$

(4) $$\mathrm{Chl}.b\;\left(\mathrm{mg}/g\right)=\frac{\left(\left(0.0229\times A_{645}\right)-\left(0.00468\times A_{663}\right)\right)\times 10}{\text{sample weight}}$$

(5) $$\mathrm{Crt}.t\;\left(\mathrm{mg}/g\right)=4.07\times A_{450}-\left(0.0435\times \mathrm{Chl}.a+0.367\times \mathrm{Chl}.b\right)$$

### 2.5 Determination of ascorbic acid content

Ascorbic acid contents of fresh and processed carrot leaves were determined using the method reported by Giovanelli et al. (2002) with some modifications. The sample was diluted 1:10 (g/mL) with 4.5% meta‐phosphoric, homogenized, and centrifuged for 10 min at 4000 *g* and 4°C. Subsequently, supernatant was filtered through a 0.45 μm syringe filter. For HPLC analyses, an ACE 5C18 (250 × 4.6 mm, ID: 5 μm) column was used with the isocratic elution of mobile phase containing ultra‐pure water (at pH 2.2 adjusted with H_3_PO_4_) at a flow rate of 0.8 mL/min at a column temperature of 30°C. The injection volume was 20 μL, and the wavelength of the detector was set to 254 nm. Results were given as “mg ascorbic acid/100 g dry matter”.

### 2.6 Color measurement

Color parameters (L*, a*, b*, C*, Hue) of the carrot leaves were measured by the colorimeter (NH310 High Quality Portable Colorimeter, Shenzhen 3NH Technology CO., LTD., China). Color measurement was conducted on the surface of dried and fresh carrot leaves at five different locations for each sample. Results were given as a mean of these five measurements for each color parameter. Color changes of samples were calculated by the following Equation (6).

(6) $$∆E=\sqrt{{\left(L^{*}-L_{0}^{*}\right)}^{2}+{\left(a^{*}-a_{0}^{*}\right)}^{2}+{\left(b^{*}-b_{0}^{*}\right)}^{2}}$$

where L*, a*, and b* are the surface color parameters of leaves. *L*_0_*, *a*_0_*, and *b*_0_* are the surface color parameters of fresh carrot leaves.

### 2.7 Statistical analyses

All experiments were conducted in triplicates for each sample. One‐way analyses of variance (ANOVA) were used by SPSS statistical software (IBM, Armonk, NY, USA). Duncan's test was performed to determine the minimum significant difference between means (*p* < .05).

## 3 RESULT AND DISCUSSION

### 3.1 Total phenolic content and total antioxidant capacity of carrot leaves

Total phenolic content (TPC) and total antioxidant activity (TAC) of fresh carrot leaves were determined as 917 ± 17 mg/100 g and 556 ± 18 mg/100 g on dry bases, respectively. The changes in TPC and TAC caused by different drying techniques and storage processes applied to carrot leaves are shown in Table 1. TPC and TAC contents of carrot leaves stored at refrigerator conditions for 7 days were found to be statistically indistinguishable from fresh carrot leaves (*p <* .05). Although the storage process at room conditions causes a significant decrease in TPC and TAC of samples, it is seen that a very high amount of TPC is still preserved. The drying process adversely affected TPC and TAC of carrot leaves. Losses in TPC and TAC both were more than 80% of their initial values when OD was used. Phenolics are heat‐sensitive compounds and have high radical scavenging ability thanks to their hydroxyl groups. A decrease in TPC during oven drying may be associated with the longer drying time under atmospheric conditions, since the presence of oxygen especially at high temperature levels causes an accelerated oxidation reaction resulting in reductions for bioactive compounds. However, it appears that the TAC of carrot leaves oven‐dried at 60°C was significantly higher than that at 40°C (*p* < .05). Polyphenols in the intermediate stage of oxidation, during drying, have more antioxidant power than the initial ones. Additionally, Maillard reaction products (MRPs) are formed at high temperatures. MRPs increase the conversion of the antioxidant activity of some bioactive compounds into more active forms, or the activation or inactivation of enzymes (e.g., oxidizing ones) (Manzocco et al., 1998; Nicoli et al., 2000; Piga et al., 2003). Similarly, it has been reported that MRPs are formed, and antioxidant activity increases with increasing temperature in Jew's mallow leaves, plums, and black tea (Mokhtar & Morsy, 2014; Piga et al., 2003; Ye et al., 2021). Among the drying processes, the least loss in TPC and TAC was observed in vacuum drying and freeze drying. Studies have shown that the vacuum drying technique causes minimal degradation in phenolic content compared to hot air drying (Karaman et al., 2014; Quintero Ruiz et al., 2014). Additionally, freeze drying is known to preserve the nutritional value of products (Rahman et al., 2015). Therefore, freeze and vacuum drying techniques are the methods that can be used when carrot leaves dry. In this study, it was determined that MW‐IR drying was superior to OD in terms of both TPC and TAC of dried carrot leaves (*p* < .05). This better protection performance of MW‐IR is thought to be related to its short drying time. Therefore, MW‐IR is a promising drying technique compared to oven drying.

**TABLE 1:** Change in total phenolic content and total antioxidant capacity of carrot leaves powder depending on drying methods and storage conditions.

	F	OD 60°C	OD 40°C	MW + IR	FD	VD 60°C	RT	R
TPC*	917 ± 17^a,**^	131 ± 9^d^	104 ± 18^d^	174 ± 5^d^	205 ± 28^c,d^	297 ± 45^c^	620 ± 35^b^	888.8 ± 62.7^a^
TAC*	556 ± 18^a^	167 ± 13^d,e^	103 ± 4^f^	119 ± 5^e,f^	246 ± 34^b,c^	216 ± 26^c,d^	303 ± 15^b^	503.4 ± 3.5^a^

Abbreviations: F, fresh carrot; FD, freeze drying; MW + IR, microwave infrared drying; OD, oven drying; R, refrigerator; RT, room temperature drying; VD, vacuum drying.

* Results are given as mg/100 g db.

Different letters in the same row are significantly different (*p* < .05).

### 3.2 Ascorbic acid content

Ascorbic acid is one of the main components responsible for antioxidant activity in green leaves and fruits (Imtiyaj Khan et al., 2011). Ajayi et al. (1980) concluded in their study that ascorbic acid constitutes almost 100% of vitamin C. Ascorbic acid content (AAC) of fresh carrot leaves was determined to be 186 ± 4 mg/100 g. El Sharaa and Mussa (2019) reported that the AAC contents of leafy vegetables such as spinach, celery, mint, arugula, and green onions are 52, 49, 39, 28, and 25 mg/100 g respectively. Additionally, the AAC content of parsley was determined to be 38 mg/100 g. In this context, it can be said that carrot leaves, which belong to the parsley family, have higher AAC than both parsley and many other leafy vegetables. Thus, carrot leaves are promising agricultural waste.

**FIGURE 1:**
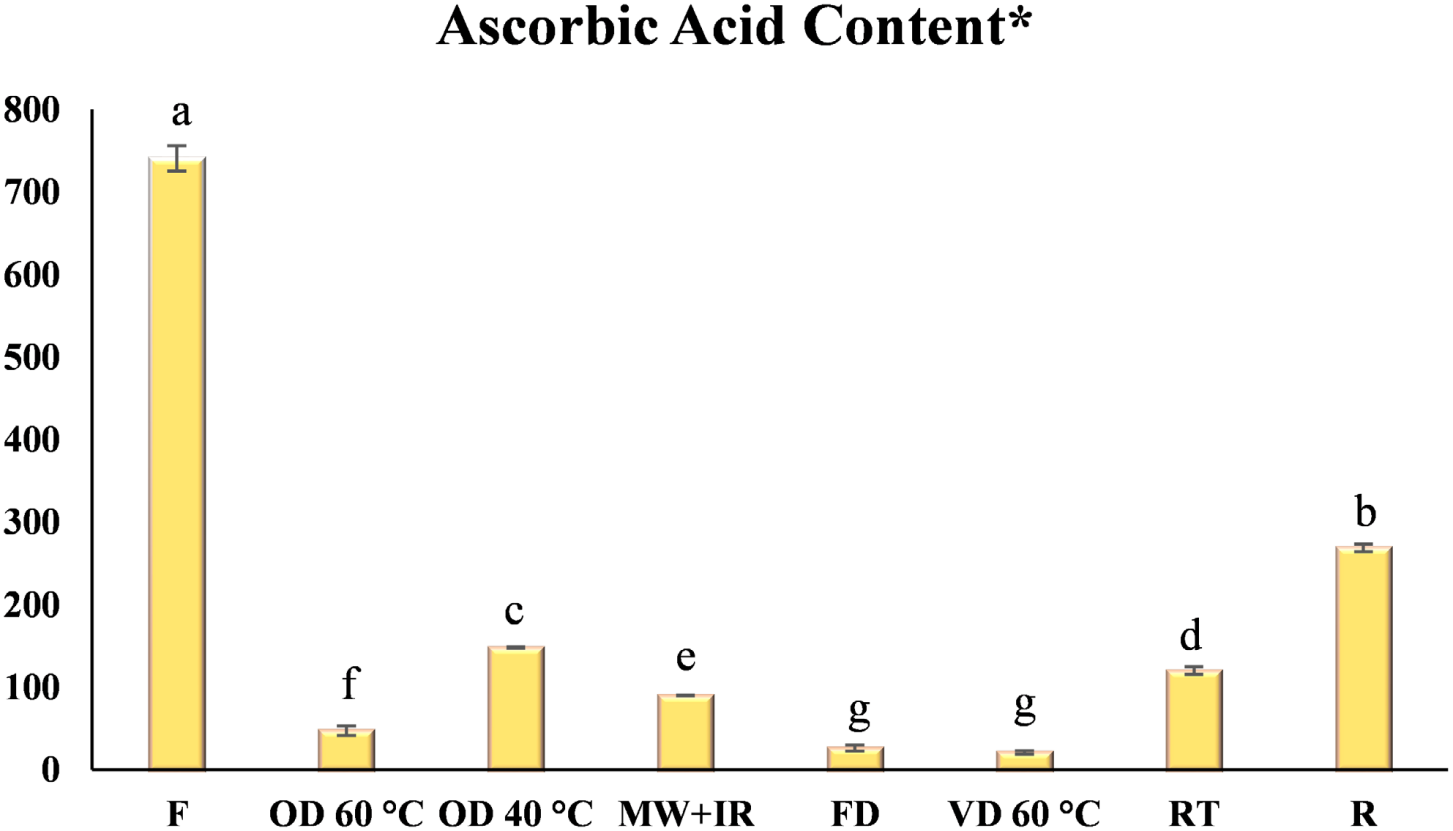
Ascorbic acid content of carrot leaves. F, fresh carrot; FD, freeze drying; MW + IR, microwave infrared drying; OD, oven drying; R, refrigerator; RT, room temperature drying; VD, vacuum drying. *Results are given as mg/100 g db. **Different letters in the figure are significantly different (*p* < .05).

Ascorbic acid content of the dried kiwifruit samples decreased significantly, irrespective of the technique used (Figure 1). According to the ascorbic acid retention of carrot leaves after drying and storage, the order from highest to lowest was R > OD40°C > RT > MW‐IR > OD60°C > FD > VD60°C (Figure 1). On the seventh day of storage, a significant decrease was detected in the ascorbic acid content of samples both in the refrigerator and at room temperature. Carrot leaves dried by different methods were also examined in terms of their AAC levels. The highest ascorbic acid value was measured in leaves that were dried in an oven at 40°C. However, an increase in temperature from 40 to 60°C for OD resulted in the highest ACC loss. It is well known that ascorbic acid is a heat‐sensitive compound and literature shows that ascorbic acid loses, especially after 60°C (Vieira et al., 2000). Chin et al. (2015) reported that the effect of temperature on ascorbic acid loss in kiwifruit dried with hot air (40, 50, and 60°C) was greater than the drying time. The AAC result of MW‐IR drying was found to be lower than OD at 40°C and higher than OD at 60°C. It is known that in microwave applications, the local temperature may reach high levels, thus AAC content is reduced more than that observed for OD at 40°C due to the adverse effect of high temperature. On the other hand, rapid processing limits this high temperature effect and produces better results for nutritional value and bioactive content of food materials. This is why AAC of carrot leaves dried by MW‐IR was higher than OD at 60°C's result. The vacuum drying method was found to adversely affect the ascorbic acid content of carrot leaves more than other studied techniques except for FD (*p* < .05). Severe loss in ascorbic acid may be attributed to the thermal degradation as well as oxidation reaction which occurs during vacuum drying. This is thought to be because of the pore size increase as a result of vacuum application which makes oxygen penetration through the leaf surface easy and results in an accelerated oxidation reaction. In the current vacuum system, the vacuum level was only 80 kPa (21 kPa absolute pressure) and this pressure level was still high to avoid the adverse effect of oxygen on carrot leaves in the drying chamber. Besides, the studied temperature level was also high for the stability of ascorbic acid (Vieira et al., 2000). Thus, this interaction effect of both process parameters (pressure and temperature) in VD could create the observed highest loss in AAC of carrot leaves.

### 3.3 Surface color values of carrot leaves

The surface color values (L*, a*, and b*) of fresh, stored, and dried carrot leaves are listed in Table 2. The initial values of the brightness (*L*_0_*), redness (*a*_0_*), and yellowness (*b*_0_*) of fresh samples were 45.1 ± 1.5, −6.6 ± 0.9, and 25.0 ± 1.1, respectively. In the current study, processes and storage periods were investigated to evaluate their effects on the surface color parameters of carrot leaves. In contrast to fresh leaves, regardless of the methods and/or conditions employed, the L* values of carrot leaves decreased by the conclusion of the drying processes (*p* < .05) except for the result of the trial of OD 40°C (*p* > .05). Similarly, brightness values of samples were adversely affected during storage (*p* < .05). The best preservation was achieved for dried samples pretreated with OD and dried at 40°C. These results indicate that storing carrot leaves at room temperature is a better choice than storage in a refrigerator. Some enzymatic and non‐enzymatic browning reactions occur in foods during processing and storage periods, which cause deterioration of quality properties. Generally, most plant tissues contain phenolics, which are substrates for a reaction controlled by a naturally occurring enzyme called polyphenoloxidase (Perera & Baldwin, 2000). Oxidized phenolic forms polymerize as a by‐product of this enzymatic activity, resulting in brown‐colored pigments after drying and storage. Additionally, the Maillard reaction (a browning event that results from the combination of reducing sugars with amino acids), caramelization, and ascorbic acid browning may occur during drying and storage (Burdurlu & Karadeniz, 2002; Perera & Baldwin, 2000). These browning reactions are responsible for the decrease in the brightness value and the increase in the redness value. In addition, the transformation of chlorophylls into pheophytins during heat treatment appears to be another reason for browning and decreasing greenness (Kamel, 2013). Chlorophyll is the main pigment that gives thermally processed vegetables a green color and is sensitive to high temperatures and long processing times hence, the degree of greenness affects the final quality of the product (Sánchez et al., 2014). The thermal process determines the amount of chlorophyll degraded. There was an increase in a* values of carrot leaf samples for all drying trials and storage periods when this value was considered for fresh leaves. Although there was a slight change in a* value for OD 40°C treatment, still the closest to that of fresh leaves. For storage trials, similar to the result observed for the brightness value, the room temperature trial is the best choice to obtain an identical a* value to a fresh one compared to the storage in the refrigerator. Negative a* values indicate that dried carrot leaves keep their green color. According to our results it is clear that negative a* values are still characteristic of dried or stored carrot leaves except for FD and VD at 60°C. Although the visualized surface color was green for the dried leaves, color changes in processed or stored carrot leaves were strong compared to fresh samples. As mentioned above, these increases in a* values of dried stored carrot leaves can be associated with enzymatic and non‐enzymatic browning reactions. On the other hand, the browning that occurs in vacuum‐dried products is due to melanoidins formed as a result of non‐enzymatic browning (Sultana et al., 2012). b* values of carrot leaves were found between 17.6 and 25.2. The highest yellowness value is in the MW + IR dried samples, as it decreases in drying processes at high temperature levels as well as in long storage periods. Chlorophylls are the main coloring pigment group in green leafy vegetables (Sánchez et al., 2014), and they are sensitive to high temperatures and long process time. Being coincidence to our results, studies have shown that high temperature levels (drying can be considered to this extent) cause the degradation of color pigments in food (Fennell et al., 2004). On the other hand, the browning that occurs in vacuum‐dried products is due to melanoidins formed as a result of non‐enzymatic browning (Sultana et al., 2012). The achievement of similar surface color values to the corresponding values of the fresh product is an important criterion for the assessment of the success of the applied process. The lowest color difference among processed and fresh samples was obtained for OD 40°C and the highest change was observed in freeze‐dried samples. These results were also observed for each color parameter, and it was concluded that OD 40°C treatment was preferable to preserve surface color of carrot leaves among the studied drying processes. For the storage period, the calculated Δ*E* values indicate that room temperature conditions are more suitable for storing fresh carrot leaves in terms of color properties than storage at refrigeration.

**TABLE 2:** Change in surface color values and total color difference of carrot leaves powder depending on drying methods and storage conditions.

	*L* *	*a* *	*b* *	*C* *	*h*°	Δ*E*
F	45.11 ± 1.52^a,**^	−6.60 ± 0.85^f^	25.01 ± 1.09^a,b^	25.90 ± 1.22^a^	104.63 ± 1.40^a^	–
OD 60°C	39.95 ± 0.33^c^	−1.42 ± 0.14^c^	21.60 ± 0.11^c^	21.65 ± 0.11^c^	93.76 ± 0.36^d^	8.09 ± 0.21^c^
OD 40°C	44.25 ± 0.58^a,b^	−4.74 ± 0.23^e^	23.81 ± 0.24^a,b^	24.28 ± 0.26^a,b^	101.24 ± 0.48^b^	2.59 ± 0.42^e^
MW + IR	41.51 ± 0.36^c^	−1.98 ± 0.14^c,d^	25.22 ± 0.35^a^	25.30 ± 0.35^a,b^	94.45 ± 0.28^d^	5.91 ± 0.33^d^
FD	31.59 ± 1.03^e^	0.01 ± 0.56^b^	17.56 ± 0.58^e^	17.60 ± 0.58^d^	89.77 ± 1.85^e^	16.80 ± 1.29^a^
VD 60°C	37.43 ± 0.66^d^	1.93 ± 0.16^a^	23.56 ± 0.47^b^	23.66 ± 0.47^b^	85.31 ± 0.36^f^	11.65 ± 0.49^b^
RT	42.10 ± 0.08^b,c^	−4.26 ± 0.07^e^	21.27 ± 0.06^c,d^	21.75 ± 0.08^c^	101.32 ± 0.20^b^	5.34 ± 0.06^d^
R	37.18 ± 0.35^d^	−2.63 ± 0.13^d^	20.06 ± 0.35^d^	20.23 ± 0.36^c^	97.45 ± 0.33^c^	10.17 ± 0.46^b^

Abbreviations: F, fresh carrot; FD, freeze drying; MW + IR, microwave infrared drying; OD, oven drying; R, refrigerator; RT, room temperature drying; VD, vacuum drying.

* Results are given as mg/100 g db.

Different letters in the same row are significantly different (*p* < .05).

### 3.4 Effect of different drying and storage conditions on chlorophyll and carotenoid contents

Carotenoids are sensitive to light and oxygen but are stable at high temperatures. In the absence of oxygen and light in the environment, carotenoids do not deteriorate if they are cooked or boiled. However, there is a decrease in the amount of chlorophyll as the heat exposure increases since the chlorophyll pigment is heat sensitive. Chlorophylls and carotenoids are common pigments that give the characteristic colors to vegetables and various fruits. It was determined that the amount of chlorophyll detected after all drying methods decreased significantly compared to fresh carrot leaves due to the increase in temperature and the related oxidation (Alibas et al., 2021). In this study, the chlorophyll *a*, chlorophyll *b*, total amount of chlorophyll, and carotenoid contents are given in Table 3. For different drying and storage conditions, chlorophyll‐*a* ranged from 9 to 22 mg/100 g db, chlorophyll‐*b* ranged from 6 to 22 mg/100 g db, and total chlorophyll content was found to be in the range of 26 to 253 mg/100 g db. In addition, for all these treatments, total carotenoid content was found to be between 4 and 25 mg/g. In all these drying treatments, total chlorophyll content was in order of OD60°C > OD40°C > FD > MW‐IR > VD60°C, and total carotenoid content was in order of OD60°C > VD60°C > OD40°C > FD > MW‐IR. Among the stored samples, the highest amount of chlorophyll and carotenoid were found in carrot leaves stored in the refrigerator. When the results were evaluated, the samples that had higher chlorophyll *a* and chlorophyll *b* values in carrot leaves and more acceptable were the samples dried under OD 60°C conditions. Similar to Saini et al. (2014), a higher retention of chlorophyll‐*a* than chlorophyll‐*b* was observed for all drying methods.

**TABLE 3:** Change in chlorophyll *a*, chlorophyll *b*, total chlorophyll, and total carotenoid contents of carrot leaves powder depending on drying methods and storage conditions.

	F	OD 60°C	OD 40°C	MW + IR	FD	VD 60°C	RT	R
Chl.a*	21.6 ± 0.1^a,**^	16.2 ± 0.20^b^	13.9 ± 0.39^c^	9.5 ± 0.4^d^	9.5 ± 0.5^d^	8.6 ± 0,12^d^	14.6 ± 0.9^c^	21.1 ± 0.83^a^
Chl.b*	22.4 ± 0.8^a^	12.6 ± 0.23^b^	9.5 ± 0.26 ^b,c,d^	6.3 ± 0.3^d^	7.1 ± 0.44 ^c,d^	6.6 ± 0,16^d^	10.3 ± 1.03 ^b,c^	19.6 ± 2.61^a^
Chl.t*	145.6 ± 2.9^b^	48.0 ± 0.8^c^	39.4 ± 1.1^c^	26.2 ± 1.1^c^	31.3 ± 1.7^c^	25.8 ± 0,5^c^	44.3 ± 3.1^c^	253.0 ± 17.3^a^
Crtn.t*	2588.9 ± 1.9^a^	625.2 ± 3.0^c^	549.7 ± 10.4^d^	364.2 ± 14.4^e^	396.1 ± 19.0^e^	413.7 ± 6,8^e^	640.7 ± 16.2^c^	2497.0 ± 29.0^b^

Abbreviations: F, fresh carrot; FD, freeze drying; MW + IR, microwave infrared drying; OD, oven drying; R, refrigerator; RT, room temperature drying; VD, vacuum drying.

* Results are given as mg/100 g db.

Different letters in the same row are significantly different (*p* < .05).

## 4 CONCLUSION

In this study, fresh carrot leaves were examined to figure out its functional potential and the results showed high amount of phenolic compounds and relevant antioxidant activity. Besides, carrot leaves were found to be a superior source of vitamin C compared to determine that many leafy vegetables and even parsley from its own family. In order to produce value‐added products from carrot leaves they were dehydrated by OD and the temperature effect was studied at two levels. Additionally, MW‐IR, FD, and VD techniques were used for leaves dehydration. The drying technique was seen to be significant in affecting the phenolic, vitamin C contents as well as antioxidant activity. According to the results, the MW‐IR technique is the superior method. In the OD method temperature change caused an adverse impact on functional constituents which decreased with a temperature increase. Considering the case of direct consumption storage of carrot leaves was also studied under two different conditions: RT and R. Results showed that carrot leaves were stored in a refrigerator for longer shelf life with better functional properties than storage at room temperature.

As a conclusion, carrot leave is a promising source of functional constituents, surprisingly including vitamin C. Thus, to utilize its potential more investigation about its processing as a value‐added product is required instead of agriculture waste. This study serves towards this aim and presents the preliminary results of drying processes which can be evaluated for future projects.

## AUTHOR CONTRIBUTIONS

**Merve Seçil Bardakçi:** Formal analysis (equal); investigation (equal); writing – original draft (equal). **Ayşe Özçelik:** Formal analysis (equal); investigation (equal); writing – original draft (equal). **Erkan Karacabey:** Investigation (equal); methodology (equal); supervision (equal); writing – original draft (equal); writing – review and editing (equal).

## CONFLICT OF INTEREST STATEMENT

The authors declare no conflicts of interest.

## Data Availability

The data that support the findings of this study are available on request from the corresponding author. The data are not publicly available due to privacy restrictions.
